# Differences in Cognitive Functioning in Two Birth Cohorts Born 20 Years Apart: Data from the Interdisciplinary Longitudinal Study of Ageing

**DOI:** 10.3390/brainsci12020271

**Published:** 2022-02-15

**Authors:** Christina Degen, Claudia Frankenberg, Pablo Toro, Johannes Schröder

**Affiliations:** 1 Section of Geriatric Psychiatry, Department of General Psychiatry, University Hospital Heidelberg, 69115 Heidelberg, Germany; claudia.frankenberg@med.uni-heidelberg.de (C.F.); johannes.schroeder@med.uni-heidelberg.de (J.S.); 2 Department of Psychiatry, Medicine School, Pontificia Universidad Católica de Chile, Santiago 8330077, Chile; ptoro@uc.cl; 3 Advanced Center for Chronic Disease, School of Medicine, Pontificia Universidad Católica de Chile, Santiago 8380492, Chile

**Keywords:** cognitive performance, Flynn effect, aging, cognitive reserve

## Abstract

We compared neuropsychological functioning and prevalence of mild cognitive impairment (MCI) in two birth cohorts born 20 years apart when participants had reached the same age, i.e., the mid-60s. The study followed up 500 volunteers born 1930–1932 (C30) and 502 born 1950–1952 (C50). Participants underwent medical, neuropsychological, and psychiatric examinations in 1993–1996 (T1), 1997–2000 (T2), 2005–2008 (T3), and 2014–2016 (T4), including assessment of abstract thinking, memory performance, verbal fluency, visuo-spatial thinking, psychomotor speed, and attention. Healthy participants from C30 at T2 (*n* = 298) and from C50 at T4 (*n* = 205) were compared using multivariate ANCOVAs. Groups slightly differed with respect to age (C50: 63.86 ± 1.14 vs. C30: 66.80 ± 0.91; *p* < 0.05) and years of education (13.28 ± 2.89 vs. 14.56 ± 2.45). After correcting for age, C50 significantly outperformed C30 in all domains except concentration and verbal fluency. After additionally adjusting for education, C50 significantly outperformed C30 in declarative memory performances and abstract thinking only. Prevalence rates of MCI were 25.2% in C30 and 9.6% in C50 (*p* < 0.001). Our findings confirm the association between better educational attainment and enhanced cognitive performance in “younger” old individuals. While this association corresponds to the Flynn effect, various life course influences may have also contributed to better performance, including improvements in healthcare provision, medication, and lifestyle factors. Their overall effects may foster cognitive reserve and thus translate into the decline in MCI prevalence reported here.

## 1. Introduction

Projections of cognitive functioning in ageing are essential for adequate organization of future healthcare provision, including the adaptation of primary and secondary preventive measures. In this respect, an increase in life expectancy may or may not go hand in hand with an increase in functional limitations and disability, including a rise in dementia prevalence. Previous comparisons demonstrated massive IQ gains over time [1]. This finding is generally referred to as the Flynn effect and was confirmed in a wealth of studies [2].

The term “intelligence” as a description of general mental abilities is not universally accepted, and different approaches to its conceptualization have been made. According to one of the earliest models, intelligence can be separated into two overlapping albeit distinct categories: fluid intelligence refers to the capacity to solve problems for which previous experience and acquired knowledge is not important, while crystallized intelligence is based on a body of knowledge that an individual has retained over time [3]. According to the results of a metanalysis based on 271 independent samples from 31 countries [2], the Flynn effect varies according to domain and primarily involve fluid followed by spatial, full-scale (fluid and crystallized IQ), and crystallized IQ test performance. “Spatial” intelligence originated in a conceptualization of multiple intelligences and refers to the ability to solve spatial problems of navigation and visualization [4].

As demonstrated in a cross-national investigation [5], the Flynn effect can at least partly be attributed to extended education. Educational achievement is generally considered the best surrogate of cognitive reserve, i.e., the ability of an individual to compensate cerebral changes, in particular due to dementing disorders such as Alzheimer’s disease (AD) and mild cognitive impairment (MCI) as its preclinical stage [6,7]. On a population level, increased cognitive reserve may at least partly compensate for the increased risk of dementing disorders due to higher life expectancy.

In the present study, we sought to investigate these findings in a prospective examination of two birth cohorts born 20 years apart. As participants were followed for more than 20 years, both cohorts could be compared in their mid-60s at nearly the same age. We expected to find performance gains in the young vs. the old cohort, independent of educational attainment. This effect should primarily involve components of fluid IQ such as executive functions and abstract thinking. Moreover, we expected that performance gains are linked to a decreased risk of mild cognitive impairment (MCI).

## 2. Materials and Methods

**Figure 1:**
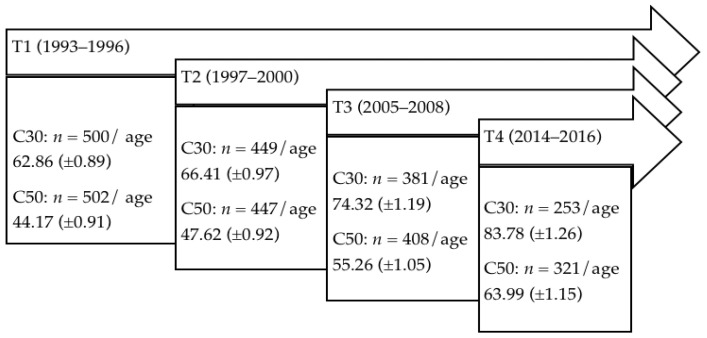
Examination waves of the Interdisciplinary Longitudinal Study of Adult Development and Aging.

Data were drawn from the Interdisciplinary Longitudinal Study of Adult Development and Aging (ILSE) that was launched in 1992. Detailed descriptions of variables and procedures of the study can be found elsewhere [8]. Select information relevant to the present study is presented below (see also Figure 1).

The ILSE is a prospective longitudinal study of two birth cohorts born 20 years apart [8]. The older birth cohort “C30” was born between 1930 and 1932, the younger birth cohort “C50” was born between 1950 and 1952. A first examination wave was conducted between 1993 and 1996 (T1) with *n* = 1002 subjects (500 participants from C30 and 502 participants from C50). A second examination was conducted between 1997 and 2000 (T2) with *n* = 896 (449 from C30 and 447 from C50). A third examination took place between 2005 and 2008 (T3) with *n* = 789 (381 from C30 and 408 from C50), and a fourth examination was conducted between 2014 and 2016 (T4) with *n* = 574 subjects (253 from C30 and 321 from C50).

For the purpose of the current study, cognitive performance of the older cohort at T2 was compared to cognitive performance of the younger cohort at T4. We opted for the second examination wave instead of the first examination wave in order to control for potential effects of repeated examinations due to test sophistication that were absent during the first examination wave.

Exclusion criteria were the presence of age-associated cognitive decline (AACD), which can be compared with multiple domain MCI [9,10]; Alzheimer’s dementia (AD); mild cognitive disorder due to medical condition (MCD); vascular dementia (VD); and current depression or alcohol/substance dependence and/or abuse. For T2, 106 (23.6%) participants from C30 were diagnosed with AACD, and 36 (8.0%) participants were diagnosed with MCD. Of the remaining subjects, three suffered from depression, and one from alcohol dependence. Furthermore, six participants who did not complete neuropsychological testing were excluded, leaving 298 subjects for analyses.

For T4, 31 (9.7%) individuals from C50 were diagnosed with AACD, one individual (0.3%) was diagnosed with AD, 21 individuals (6.5%) suffered from MCD, and no subject was diagnosed with VD. Eight of the remaining subjects were diagnosed with depression, four with alcohol/substance dependence, and one with both depression and alcohol substance dependence, leaving 255 individuals. Fifty participants who did not fully take part in neuropsychological testing were also excluded, leaving 205 subjects for analyses.

At each examination wave, physical and mental health was assessed by a trained physician. To assess cognitive capacity, we administered different neuropsychological tests, including the subtests Word List (WL) and Digit Symbol Test (DST) of the Nuremberg Age Inventory (NAI) [11], the subtests Mosaic Test (MT) and Finding Similarities (FS) of the Wechsler Intelligence Test Battery (HAWIE-R) [12], the subtests Word Fluency (WF) and Visual Thinking (VT) of the Performance Evaluation System (Leistungsprüfsystem) [13], and the Attentiveness Endurance Test “d2” [14]. Further methodological details are described in [8,15].

All statistical analyses were performed using SPSS (version 27). For significance tests, an alpha of 0.05 was set, such that *p*-values less than 0.05 were considered significant. For descriptive analyses, ANOVAs were used. In the case of categorical variables, group differences were computed with chi-squared tests. To examine cohort differences in cognitive aging multivariate ANCOVAs were calculated. While the respective cognitive test scores (WL, DST, MT, FS, WF, VT, d2) were dependent, the cohort was the independent variable. To address potential effects of educational achievement and minor age differences between the cohorts, we included years of school education and age as covariates. Higher educational achievement may also involve a greater study adherence. Potential selection effects were addressed by comparing years of formal school education of the entire sample with the subsample considered in the present analysis. To examine the proportion of persons with MCI, we compared the prevalence rates of MCI in C30 at T2 and C50 at T4 using a chi-squared test.

## 3. Results

A comparison of demographic characteristics can be seen in Table 1. Participants in C50 were slightly younger at T4 than participants of C30 at T2 and had undergone more years of education. The ratio of male/female participants was comparable between cohorts.

Neuropsychological performance and results of the multivariate ANOVAS and ANCOVAs are presented in Table 2. Accordingly, the young cohort showed significantly increased performance levels in all neuropsychological domains except for concentration and verbal fluency. These differences remained stable after adjusting for age. A significant main effect of education was found for all conducted tests except “word list immediate recall”. After correcting for age and education, we found that a significant main affect arose for declarative memory (“WL recall” and “WL delayed recognition”) and abstract thinking (“MT”).

Prevalence of MCI was 23.6% in the earlier-born (C30) and 9.7% in the later-born (C50) cohort. Chi-squared tests revealed a significantly higher MCI rate for C30 than for C50 (*χ^2^*(1) = 24.91, *p* < 0.001).

To identify potential selection effects, we compared school education of the two cohorts under examination with the respective values obtained in the original cohorts at study intake. As to be expected, both cohorts investigated here showed a slightly higher number of educational years when compared with the original cohort at intake (C30_years of education_: 13.32 at T2 vs. 12.45 at T1; C50_years of education_: 14.69 at T4 vs. 13.60 at T1). These differences reached the significance level (C50: *F*(1, 500) = 23.83, *p* < 0.001; C30: *F*(1, 498) = 18.38, *p* < 0.001).

## 4. Discussion

The present study yielded two major findings: (i) further support of an increased neuropsychological performance in younger birth cohorts partially due to greater educational achievement as predicted by the Flynn effect, and (ii) an indication that this effect may translate into a lower risk of MCI in the mid-60s.

When compared with the old birth cohort, the young cohort was characterized by better educational attainment, as demonstrated by a significantly higher number of years of education. Both cohorts comprised similar proportions of women and men and showed only minor—albeit marginally significant—age differences. Hence, age was included as a covariate in all analyses.

Along with that, the young outperformed the old cohort in all neuropsychological domains examined, except concentration and verbal fluency. A significant effect of education was confirmed for all domains except memory (word list free recall). When adjusted for age and education, the differences in declarative memory performance (word list immediate and delayed recall) and abstract thinking (mosaic test) remained solely significant between birth cohorts, indicating that performance gains in these domains cannot be attributed to education alone.

On basis of data derived from studies in 14 nations, Flynn [1] demonstrated “massive IQ gains” in a single generation. Cohort effects were described by Kuhlen [16] as early as 1940 and were attributed to a variety of influences associated with changes in the social and cultural environment, such as quantity and quality of education or of healthcare. The meta-analysis of Pietschnig and Voracek [2] confirmed these finding and demonstrated vast annual IQ gains for fluid (0.41 IQ points/a), spatial (0.30 IQ points/a), full-scale (0.28 IQ points/a), and crystallized (0.21 IQ points/a) domains. On basis of this differentiation of IQ gains between domains, Pietschnig and Voracek [2] (p. 2) assumed “factors associated with life history speed” to be “mainly responsible for the Flynn effect”.

Different conceptualizations of “intelligence” and overlapping features thereof render a clear allocation of the neuropsychological functions investigated in our study to any intelligence framework negligible. However, for the purpose of comparison, our data suggest that general performance gains appear across different facets of “intelligence”, while differences that are independent of age and education (declarative memory and abstract thinking) can be subsumed under the terms “crystallized” [3] or “linguistic”/”logical-mathematical” intelligence [4], while performance gains with respect to “fluid” or “spatial” intelligence were associated with years of education (e.g., executive functioning, visuospatial thinking, concentration).

Using data from the German Socio-Economic Panel, Steiber [17] (p. 1) replicated a “highly significant Flynn effect” in an older population aged 50–90. Further evidence that these findings also apply to old age comes from Christensen et al. [18] who compared cognitive functioning in two Danish birth cohorts of nonagenarians born in 1905 (*n* = 2262, mean age 93.1 ± 0.3) and 1915 (*n* = 1584, mean age 95.3 ± 0.3). Members of the later-born cohort performed better than members of the earlier-born cohort in both cognitive functioning and activities of daily living suggesting a clear improvement in cognitive functioning in line with the Flynn effect. This effect applied to both women and men and was illustrated on the Mini Mental Status Examination MMSE [19] as well as a cognitive composite score. In contrast, the comparison of MMSE scores obtained in two cohorts of centenarians born 10 years apart [20] revealed only minor, non-significant differences.

In a seminal paper, Gerstorf and colleagues [21] compared age-related and mortality-related cognitive decline in an earlier- (1886–1913) and a later (1914–1948)-born cohort from the Seattle Longitudinal Study. Longitudinal analyses yielded “less steep rates of cognitive aging in all domains” [21] (p. 1026) considered between 50 and 80 years, favoring the younger cohort, which was attributed to improvements of the educational system and general health. In contrast, the consequences of terminal decline showed only little variation between cohorts.

In addition, the present study yielded a significantly reduced risk of MCI in the later- when compared to the earlier-born cohort. A similar finding was reported by Langa et al. [22], who compared prevalence rates of cognitive impairment in U.S. adults aged 70 and older. While 12.2% of the population was classified as cognitively impaired in 1993, this applied to only 8.7% of individuals in 2002. The later-born cohort exhibited more years of education, had higher net worth, had fewer IADL limitations, but had higher rates of cardiovascular risk factors and cardiovascular disease. While Langa et al. [22] did not apply a formal classification, cognitive impairment as operationalized in their study corresponded to MCI multiple domains in many facets, in particular with regards to the number of cognitive domains considered.

MCI is associated with an increased risk of developing dementia and can be thus considered as the consequence of cognitive aging. Hence, the reduced risk of MCI in the later-born cohort may result from less steep rates of cognitive aging described by Gerstorf and colleagues [21]. According to this hypothesis, one may expect the development of MCI to be postponed in the later-born cohort.

Selection effects must be discussed as a potential confounding variable. As to be expected, both cohorts investigated here showed a slightly higher number of educational years when compared with the original cohort at intake. Although these differences reached significance, the selection effect refers to a similar magnitude of the change in both groups (one additional year of education compared to the values obtained originally at study intake). In addition, potential training effects need to be considered when interpreting the results of this study. While the younger cohort did not undergo the entire neuropsychological test battery at T1 and T2, three subtests (DST, d2, word list) were conducted at all four examination waves, while other subtests (VT, FS, MT) were conducted at T1, T3, and T4 for C50. As a result, participants from C50 had undergone the testing procedure three or four times, while C30 had been tested only twice at the relevant examination waves. To address the effect of test sophistication, we opted to consider T2 rather than T1 for the older cohort. Moreover, potential training effects, in terms of an economization of cerebral activation under performance gain consolidation [23] are rather unlikely to play a role, given the length of time intervals (4 to 6 years between examination waves) and the frequency of “training” to occur (twice to four times). However, there is no conclusive research on the effect of training characteristics and individual factors, which are likely to influence the magnitude of such training effects. Differences in repeated assessment must therefore be considered as a potential confounding factor when interpreting the results of our study.

Others [24] have challenged the Flynn effect, as substantial gains in test scores had substantially plateaued by the 1990s. A potential explanation of such plateaus could be a lack of change in compound scores that mask different and possibly opposite trends in separate abilities. This hypothesis was supported by Gerstorf et al. [21], who identified number ability as the only measure that did not show a positive cohort effect. This finding confirmed previous results of the Seattle Longitudinal Study, which demonstrated that number skills reached a peak with the 1924 birth cohort and declined in later born cohorts.

The decreased MCI prevalence in the later-born cohort may either refer to decelerated cognitive aging or a lower MCI risk per se; a question that clearly calls for an additional examination wave. Educational attainment does not only contribute to cognitive reserve but also involves social differences and diverse life course influences that may have also contributed to better performance in the later-born cohort, including improvements in healthcare provision; adequate medication of age-association concomitant conditions (e.g., hypertension, hypercholesterinemia); and factors loosely labelled lifestyle that are known associates of better cognitive aging such as social engagement, physical activity, cognitively effortful occupational and leisure pursuits, and continuing education [24].

These findings add to a rather optimistic picture of cognitive aging in later-born cohorts. This development cannot be solely assigned to the beneficial effects of rising levels of education, but also refers to technological advances with increasing cognitive challenges in both, work, and leisure activities [21], as well as an overall increase in physical and mental health, by which the occurrence of cognitive impairment either decreases or is being postponed to older ages [25]. It is generally accepted that many of the respective variables also contribute to cognitive reserve [6]. However, school education is still generally considered to be the best surrogate marker of both beneficial secular effects and cognitive reserve. From a clinical perspective, their overall effects may foster cognitive reserve and thus translate into a decline in prevalence and/or delayed onset of conditions associated with cognitive decline, i.e., Alzheimer’s dementia and MCI. Understanding these benefits may facilitate our understanding of cognitive aging and the development of preventive measures.

## 5. Conclusions

We identified enhanced cognitive functioning in a younger compared to an older birth cohort, which could partially be attributed to greater educational achievement and is likely to reflect other lifestyle factors that foster cognitive reserve. In line with this, we found lower rates of MCI in the younger as opposed the older cohort when they had reached their mid-60s. Albeit painting a positive picture of cognitive ageing, further research is needed to establish whether our findings reflect a deceleration of cognitive aging or a lower risk for the development of dementia-related disorders per se.

## Figures and Tables

**Table 1:** Demographic characteristics of the sample.

	C30 (T2) *n* = 298	C50 (T4) *n* = 205	*χ^2^/F*(df)	*p*
Sex (m/f)	150/148	107/98	*χ^2^*(1) = 1.68	=0.68
Age in years (SD)	66.80 (±0.91)	63.86 (±1.14)	*F*(1, 501) = 31.79	<0.001
Education in years (SD)	13.32 (±2.89)	14.69 (±2.44)	*F*(1, 501) = 1032.46	<0.001

**Table 2:** Neuropsychological performance in the earlier- (C30) vs. the later (C50)-born cohorts.

Domain Subtest	C30 (T2)	C50 (T4)	*F*_cohort_ (Uncorrected ◊)	*F*_cohort_ (Corrected ◦)	*F*_education_	*F*_age_
Declarative memory						
WL recall	5.36 ± 1.47	5.83 ± 1.46	*F*(1, 501) = 12.57 ***	*F*(1, 499) = 3.93 *	*F*(1, 499) = 3.15	*F*(1, 499) = 0.81
WL delayed recognition	7.39 ± 2.31	8.38 ± 2.12	*F*(1, 501) = 23.53 ***	*F*(1, 499) = 10.25 **	*F*(1, 499) = 4.58 *	*F*(1, 499) = 0.86
Executive functioning						
DST	45.44 ± 10.01	48.92 ± 9.07	*F*(1, 501) = 15.66 ***	*F*(1, 499) = 0.98	*F*(1, 499) = 47.33 ***	*F*(1, 499) = 0.25
Abstract thinking						
MT	25.94 ± 8.12	32.73 ± 8.32	*F*(1, 501) = 83.38 ***	*F*(1, 499) = 7.58 **	*F*(1, 499) = 75.88 ***	*F*(1, 499) = 3.65
FS	25.86 ± 4.77	26.90 ± 3.02	*F*(1, 501) = 7.74 ***	*F*(1, 499) = 0.01	*F*(1, 499) = 131.15***	*F*(1, 499) = 0.16
Visuospatial thinking						
VT	22.00 ± 6.24	25.12 ± 5.80	*F*(1, 501) = 32.18 ***	*F*(1, 499) = 0.13	*F*(1, 499) = 86.97 ***	*F*(1, 499) = 9.74 **
Concentration						
“d2“	152.30 ± 36.69	152.24 ± 28.68	*F*(1, 501) = 0.00	*F*(1, 499) = 3.70	*F*(1, 499) = 77.00 ***	*F*(1, 499) = 0.67
Verbal fluency						
WF	32.69 ± 8.74	32.91 ± 8.20	*F*(1, 501) = 0.08	*F*(1, 499) = 1.71	*F*(1, 499) = 40.29 ***	*F*(1, 499) = 0.48

◊ without adjusting for education and age; ◦ after adjusting for education and age; *** indicate statistically significant differences with * *p* < 0.05, ** *p* < 0.01, *** *p* < 0.001.

## Data Availability

The data presented in this study are available on request from J.S.
